# Identification of key genes involved in type 2 diabetic islet dysfunction: a bioinformatics study

**DOI:** 10.1042/BSR20182172

**Published:** 2019-05-31

**Authors:** Ming Zhong, Yilong Wu, Weijie Ou, Linjing Huang, Liyong Yang

**Affiliations:** 1 Endocrinology Department, The First Affiliated Hospital of Fujian Medical University, Fuzhou 350005, Fujian, China; 2 Liver Research Center, The First Affiliated Hospital of Fujian Medical University, Fuzhou 350005, Fujian, China; 3 Diabetes Research Institute of Fujian Province, Fuzhou 350005, Fujian, China

**Keywords:** bioinformatics, DEGs, GEO, GSEA, type 2 diabetes

## Abstract

**Aims**: To identify the key differentially expressed genes (DEGs) in islet and investigate their potential pathway in the molecular process of type 2 diabetes.

**Methods**: Gene Expression Omnibus (GEO) datasets (GSE20966, GSE25724, GSE38642) of type 2 diabetes patients and normal controls were downloaded from GEO database. DEGs were further assessed by enrichment analysis based on the Database for Annotation, Visualization and Integrated Discovery (DAVID) 6.8. Then, by using Search Tool for the Retrieval Interacting Genes (STRING) 10.0 and gene set enrichment analysis (GSEA), we identified hub gene and associated pathway. At last, we performed quantitative real-time PCR (qPCR) to validate the expression of hub gene.

**Results**: Forty-five DEGs were co-expressed in the three datasets, most of which were down-regulated. DEGs are mostly involved in cell pathway, response to hormone and binding. In protein–protein interaction (PPI) network, we identified ATP-citrate lyase (ACLY) as hub gene. GSEA analysis suggests low expression of ACLY is enriched in glycine serine and threonine metabolism, drug metabolism cytochrome P450 (CYP) and NOD-like receptor (NLR) signaling pathway. qPCR showed the same expression trend of hub gene ACLY as in our bioinformatics analysis.

**Conclusion**: Bioinformatics analysis revealed that ACLY and the pathways involved are possible target in the molecular mechanism of type 2 diabetes.

## Introduction

According to International Diabetes Federation (IDF) reports, there were 425 million diabetic patients worldwide in 2017, of which type 2 diabetes accounted for more than 90%. It is estimated that in 2045 the number of people with diabetes will increase to 629 million [1]. Diabetes often leads to cardiovascular complications, which have a serious impact on the patient’s life and quality of life, and also cause a heavy social burden. The pathophysiological mechanism of type 2 diabetes is still not very clear at present. It is currently believed that type 2 diabetes is mainly caused by genetic factors and the environmental influence [2,3]. There have been many basic researches on the pathogenesis of diabetes. Type 2 diabetes related diagnosis and treatment have progressed year by year recently, but the incidence of diabetes is still increasing [4]. One of the reasons may be that most studies were performed on cells and animals but not humans and were not targeted at a particular genetic event. The genetic cause accounts for a part of the etiology in type 2 diabetes. To prevent and reduce the complications of type 2 diabetes, it is especially important to clarify the pathophysiological mechanism at genetic level.

At present, with the wide use and development of high-throughput sequencing, bioinformatics analysis is of great advantage for understanding the pathophysiological mechanism of type 2 diabetes on the genetic basis. Many bioinformatics datasets in Gene Expression Omnibus (GEO) database (https://www.ncbi.nlm.nih.gov/geo/) have been applied in mining the pathogenesis of type 2 diabetes. A research revealed that the methylation status of key genes participate in the pathogenesis of diabetic nephropathy [5]. Studies have explored mRNA expression profiles in the skeletal muscle, retina and peripheral blood of type 2 diabetes in GEO [6–8]. Islet is an important organ involved in the type 2 diabetes, and studies have demonstrated previously bioinformatics analysis in islet GEO datasets. However, no study was designed to integrate all the islet datasets in GEO. In the present study, we integrated all the three islet datasets of human type 2 diabetes datasets in GEO, using R software packages and bioinformatics analysis to explore molecular mechanism of the pathogenesis in diabetic islet.

## Materials and methods

### Datasets

GEO Datasets database was selected for our study. We used the key words ‘diabetes mellitus, type 2’[MeSH Terms] OR type 2 diabetes [All Fields]) AND (‘gds’[Filter] AND ‘human’[Organism] AND ‘Expression profiling by array’[Filter]). Next, we screened these datasets according to the following inclusion criteria: (i) islet tissues; (ii) normal islets used as controls. The gene expression datasets of GSE20966, GSE25724 and GSE38642 were included. The platform for GSE20966 is GPL1352, [U133_X3P] Affymetrix Human X3P Array, which includes ten samples of β-cells obtained from normal pancreases and ten samples of pancreases β-cells obtained from two diabetic subjects. The platform for GSE25724 is GPL96, [HG-U133A] Affymetrix Human Genome U133A Array, which includes seven normal pancreatic islets samples and six type 2 diabetes pancreatic islets samples. The platform for GSE38642 is GPL6244, [HuGene-1_0-st] Affymetrix Human Gene 1.0 ST Array [transcript (gene) version], which includes 54 normal pancreatic islets samples and 9 type 2 diabetes pancreatic islets samples. MINiML formatted family file(s) were downloaded.

### Screening of differentially expressed genes

The downloaded platform and MINiML files were transformed by R language software 3.4.4. The probe name in the MINiML files was converted into gene symbol by the R package and saved as a TXT file. Then, three datasets were standardized by quantiles. Gene differential analysis was conducted using the limma R package. We considered differentially expressed genes (DEGs) as fold change (FC) > 1.2 and *P*-value <0.05. Heatmaps were generated by a web tool ClustVis [9].

### DEGs pathway encrichment analyses

On the basis of the DEGs from the three datasets, gene ontology (GO) and Kyoto Encyclopedia of Genes and Genomes (KEGG) annotations were performed using the Database for Annotation, Visualization and Integrated Discovery (DAVID) 6.8 (https://david.ncifcrf.gov/). GO analysis is a widely useful bioinformatics tool to investigate the annotation of gene and proteins. It can be used to integrate annotation data and provide tools access to all the data provided by the project. KEGG is able to integrate currently known protein interaction network information. The pathways of KEGG mainly include: metabolism, genetic information processing, environmental information related processes, cell physiological processes and drug research. DAVID can perform biological analyses of genes. It is a comprehensive online program for interpretating biological functional annotations. A *P*-value of <0.05 was identified as significant difference.

### Protein–protein interaction program analysis

Search Tool for the Retrieval Interacting Genes (STRING) 10.0 (http://string-db.org/) is an online software of interactions of genes and proteins. Cytoscape 3.6.0 is an open-source tool for network visualization of genes and proteins. Protein–protein interaction (PPI) of the DEGs were constructed from the STRING database and were visualized by Cytoscape. Venn diagrams were performed to integrate the top 100 genes from the three GEO datasets by the website Venny (http://bioinfogp.cnb.csic.es/tools/venny/index.html). The cut-off criteria were a combined score <0.15 and a node degree of ≥10 for screening hub genes. The Molecular Complex Detection (MCODE; version 1.31) app in Cytoscape was used to analyze PPI network modules. BINGO (The Biological Networks Gene Ontology tool; version 3.03) plugin in Cytoscape was conducted to the GO network of hub genes from the PPI network.

### Gene set enrichment analysis

We selected hub gene for further Gene Set Enrichment Analysis (GSEA). GSEA is a free chip data analysis tool. It is based on the existing gene sets. GSEA performs biological information from another view. It can further improve our understanding of relevant biological events. The associations between hub gene and previously defined gene sets were analyzed. The biologically defined gene sets were obtained from the Molecular Signatures Database v5.2 (http://software.broadinstitute.org/gsea/msigdb/index.jsp). We considered normal *P*-value <0.05.

### *In vitro* glucotoxicity model

Mouse islet β cell line βTC6 cell was purchased from Shanghai Institutes for Biological Sciences, CAS (Shanghai, China). The cells were cultured in DMEM (Gibco) containing 10% (vol./vol.) FBS (PAN) at a condition of 5% CO_2_ at 37°C. The experiment was continued when the cell growth reached 70–80% confluence. The experimental cells were 6–8 passage cells. Cells were divided into two groups: normal group and glucotoxicity model group. Normal group was cultured at a concentration of 5.5 mM glucose. Glucotoxicity model group was first cultured at 5.5 mM glucose for 24 h and then incubated with 25 mM glucose for 72 h [10].

### Quantitative real-time PCR

We selected key gene for validation using quantitative real-time PCR (qPCR). Total RNA from the cells was extracted with Quick-RNA™ Microprep Kit (Zymo, California, U.S.A.). cDNA synthesis was carried out using PrimeScript™ RT Reagent Kit with gDNA Eraser (TaKaRa) according to the manufacturer’s protocol. qPCR was performed with TB Green Premix ExTaq II (Tli RNaseH Plus) (TaKaRa) and analyzed with LightCycler® 96 Instrument (Roche, Switzerland). The ΔΔ*C*_t_ method was used to calculate relative changes in mRNA and β-actin was used as the internal standard [11]. Mouse genes and primers: ATP-citrate lyase (ACLY), forward, 5′-AGGAAGTGCCACCTCCAACAGT-3′, reverse, 5′-CGCTCATCACAGATGCTGGTCA-3′; β-actin, forward, 5′-GCCACCAGTTCGCCATGGAT-3′, reverse, 5′-GCTTTGCACATGCCGGAGC-3′.

### Statistical analysis

Statistical analysis was performed on SPSS 15.0 software. Data are expressed as mean ± S.D. Student’s *t*test was used to evaluate the statistical significance of difference in two groups. *P*-value less than 0.05 was considered as significant.

## Results

### Identification of DEGs between DM and normal islet tissues

**Figure 1:**
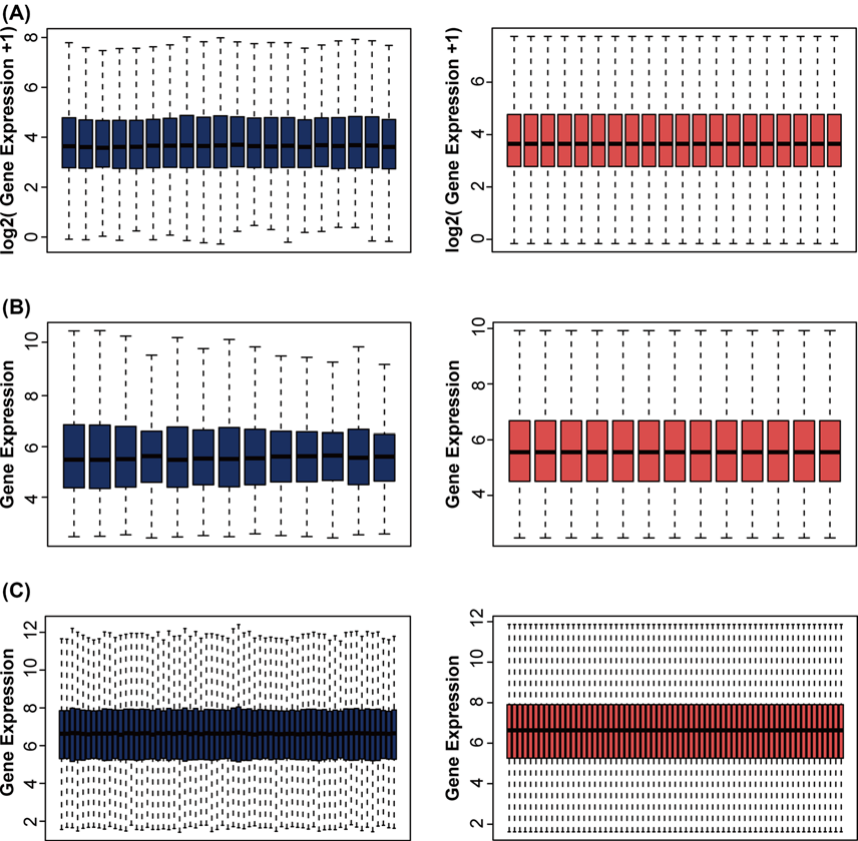
Standardization of gene expression (**A**) Standardization of GSE20966, (**B**) standardization of GSE25724, (**C**) standardization of GSE38642. The blue bar represents the data before normalization, and the red bar represents the data after normalization.

The three datasets were standardized and the results are shown in Figure 1.

**Figure 2:**
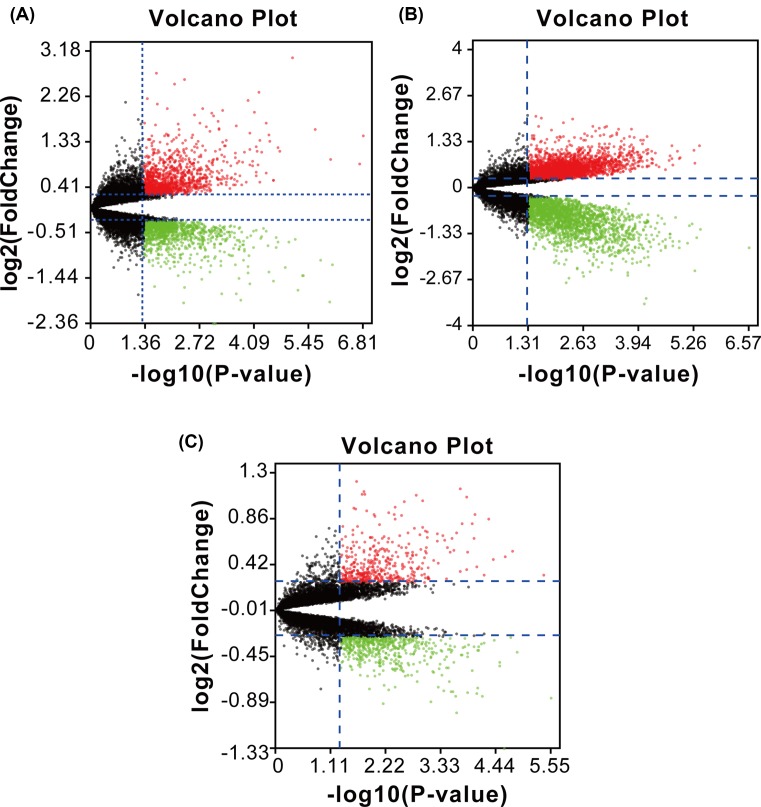
Volcano plots of DEGs between type 2 diabetes and normal control (**A**) GSE20966, (**B**) GSE25724 and (**C**) GSE38642. Data points in red represent up-regulated and green represent down-regulated genes. Genes without any significant difference are in black. The differences are set as |FC| > 1.2 and corrected *P*-value <0.05.

**Figure 3:**
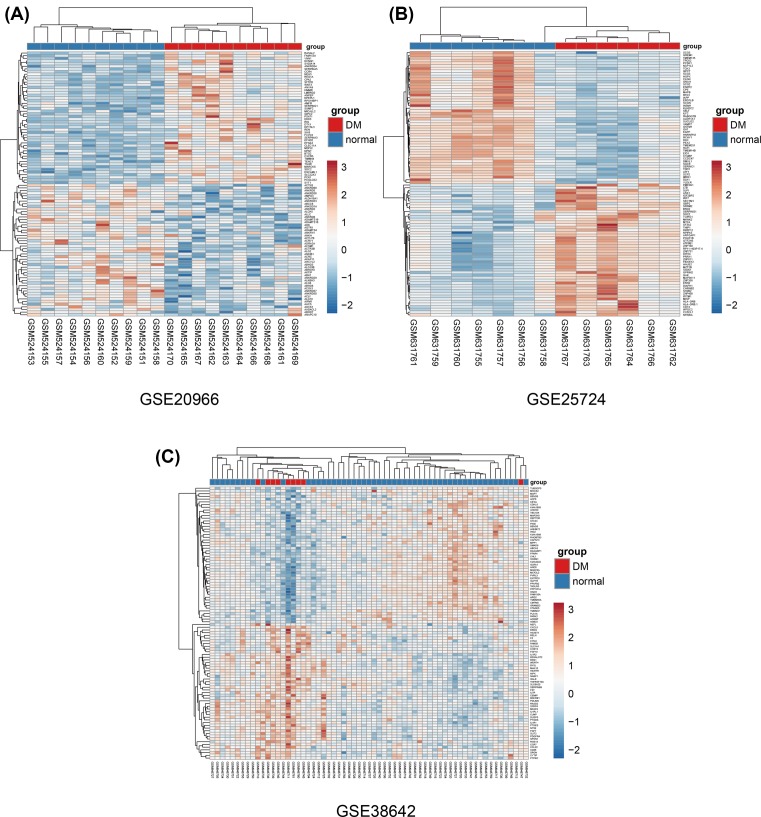
Heatmap of the top 100DEGs Heatmap of DEGs identified in (**A**) GSE20966, (**B**) GSE25724 and (**C**) GSE38642. Genes up-regulated are in red. Genes down-regulated are in blue. Genes without any significant difference are in white. The differences are set as |FC| > 1.2 and corrected *P*-value <0.05.

Then, we deleted duplicated genes and values lacking specific gene symbols. A total of 2536 DEGs were obtained in GSE20966. Among these DEGs, 1243 genes were up-regulated and 1293 genes down-regulated. Additionally, 5148 DEGs were obtained from GSE25724; there were 2992 up-regulated genes and 2156 down-regulated genes. In GSE38642, 932 DEGs were obtained. Among them, 413 were up-regulated and 519 were down-regulated. The DEGs from each dataset are shown in Figure 2. We used Euclidean distance to perform clustering. The top 100 DEGs performed by heatmap are shown in Figure 3.

### Integration of DEGs

**Figure 4:**
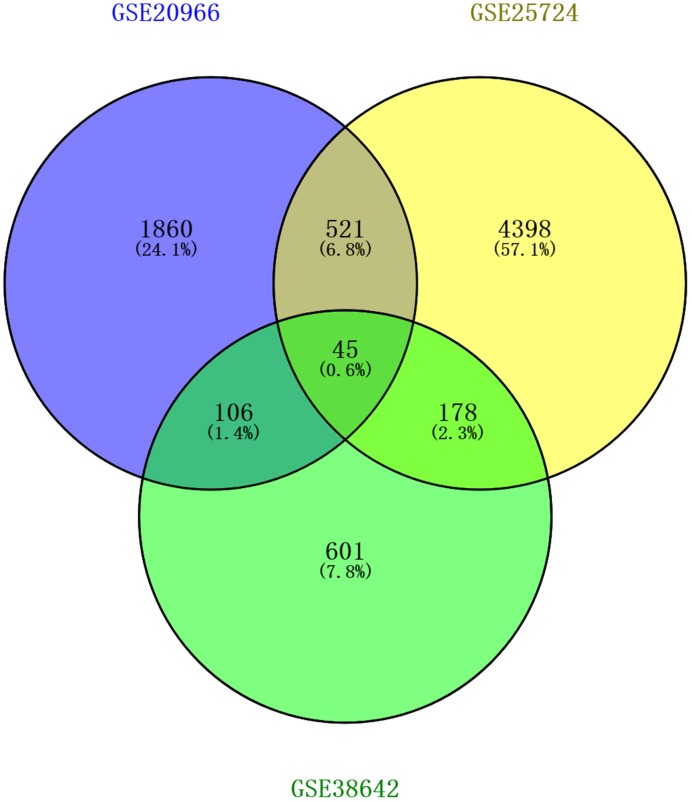
Venn diagram of common DEGs from the three datasets Forty-five DEGs were identified as common DEGs.

DEGs from the three datasets were selected for Venn diagram. The intersection of the three datasets is shown in Figure 4. A total of 45 DEGs were co-expressed in the three datasets. Among them, *APOBEC, FKBP2, GRHL2, ITGA3, PID1, PTGES, S100A14, S100A6, SAMSN1* and *THBS2* were up-regulated; while *ABAT*, α/β hydrolase domain 10 (*ABHD10*), *ACLY, ARG2, ARHGEF9, ATRNL1, BEX4, CCL21, CLGN, CTNNA2, DHRS2, EDN3, ENTPD3, GRAMD3, HADH, HLF, IAPP, KAT2B, KCNJ6, MAK16, MPP1, NBEA, NKX2-2, NOC3L, NR0B1, NRCAM, RHOBTB3, RRAGD, SEMA5A, SLC2A2, SORL1, SRD5A1, TGFBR3, TPD52* and *TRIM37* were down-regulated.

### GO biological process analysis and KEGG pathway enrichment

**Figure 5:**
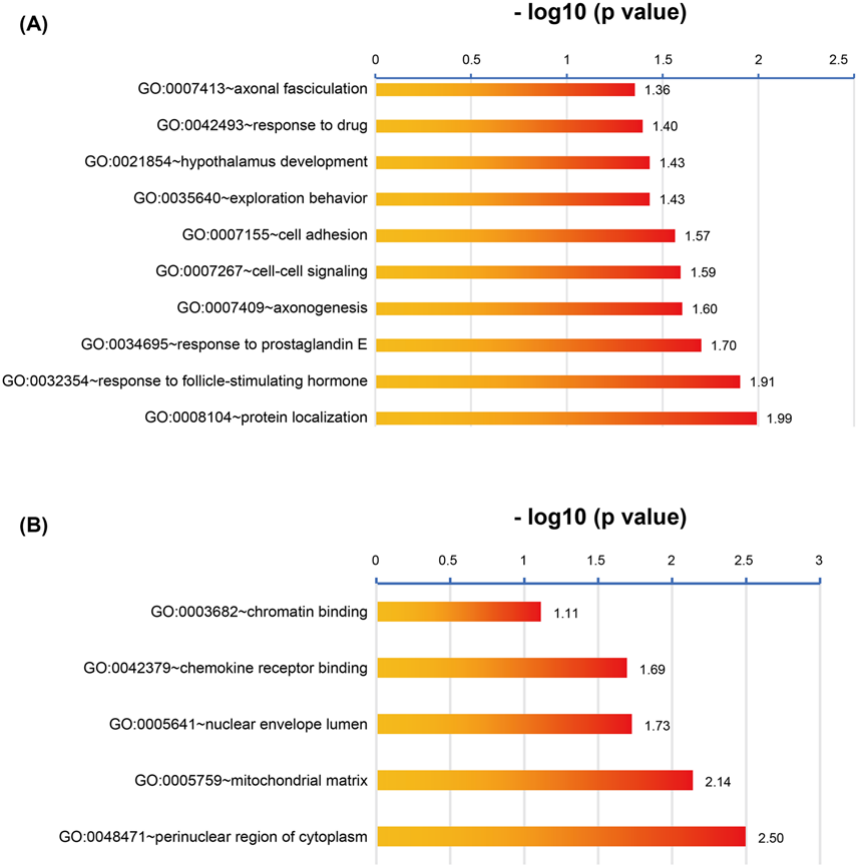
Functional analysis in GO (**A**) Biological process in common DEGs. (**B**) Molecular function in common DEGs.

GO analysis of genes include molecular function, biological processes and cell composition. In our study, GO analysis was used to perform the functional process of the DEGs. The co-expressed DEGs were carried out by the DAVID database. A *P*-value <0.05 was defined to identify up- and down-regulated genes in GO functional enrichments. The results are shown in Figure 5. GO biological process analysis found that DEGs were mainly enriched in protein localization, response to follicle stimulating hormone (FSH), response to prostaglandin E, axonogenesis and cell–cell signaling. In the cell composition part, the DEGs were involved in perinuclear region of cytoplasm, mitochondrial matrix, nuclear envelope lumen and chemokine receptor binding. In the molecular function section, the genes participated in chemokine receptor binding. The results suggested that DEGs were mostly involved in cell pathway, response to hormone and binding.

We also performed our KEGG pathway analysis. With DAVID mentioned previously, DEGs were enriched in Maturity onset diabetes of the young (MODY) (*P*<0.001). The result indicated that MODY and type 2 diabetes share some same molecular pathway.

### PPI network integration

**Figure 6:**
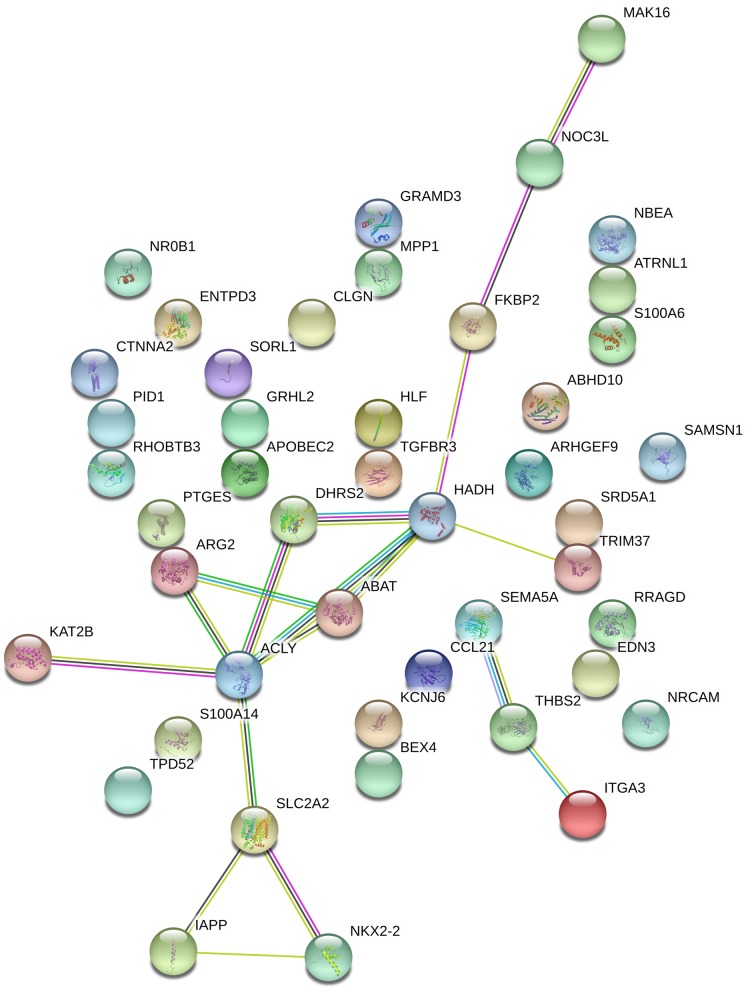
PPI network of common DEGs The balls represent the gene nodes, the connecting lines represent the interactions.

We used the STRING database (https://string-db.org) to investigate PPI networks, including 10 up-regulated genes and 35 down-regulated genes. A PPI network of DEGs was performed as shown in Figure 6. As mentioned before, we identified ACLY as hub gene (node degree of ≥10) .

**Figure 7:**
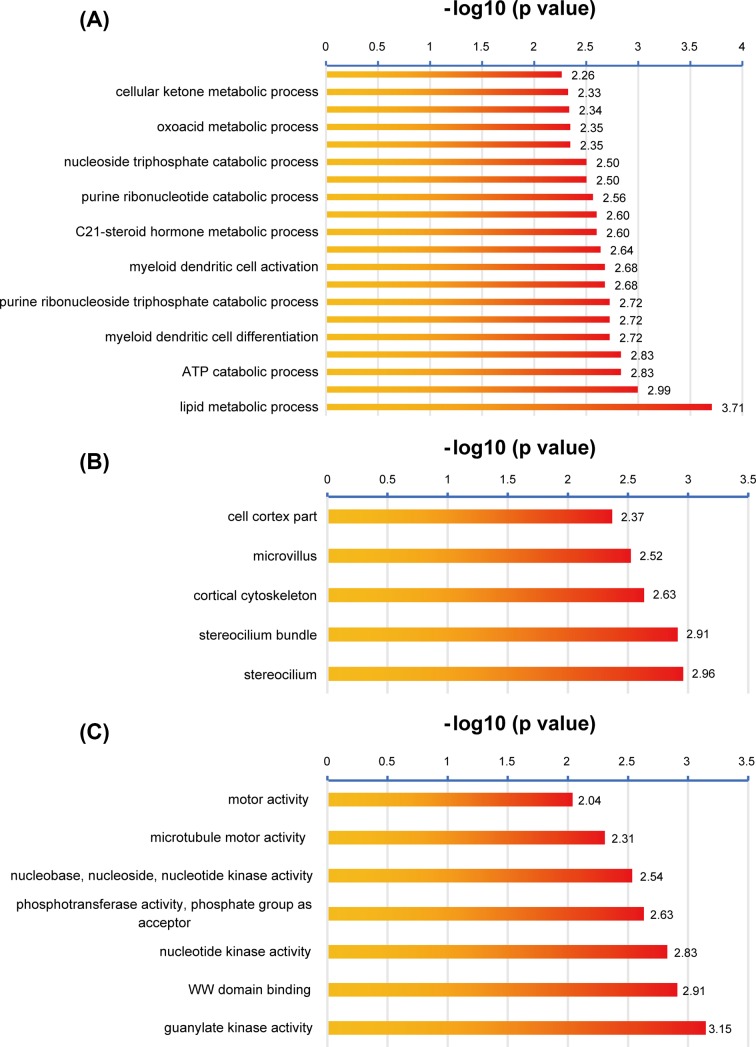
Functional enrichment by BINGO (**A**) Biological process of identified module by BINGO. (**B**) Cell composition of identified module by BINGO. (**C**) Molecular function of identified module by BINGO.

A significant module containing four nodes and six edges was constructed by MCODE. Nodes were dehydrogenase/reductase 2 (DHRS2), ABHD10, HADH, ACLY. All genes in the module were down-regulated. Then, we performed BINGO in the Cytoscape to further investigate the possible pathway of this module. Results are shown in Figure 7. The GO biological process, cell composition and molecular function were generated by BINGO. In biological process category, the top five processes by which the genes significantly enriched were lipid metabolic process, small molecule catabolic process, ATP catabolic process, citrate metabolic process and myeloid dendritic cell differentiation. In cell composition category, the genes were involved in stereocilium, stereocilium bundle, cortical cytoskeleton, microvillus and cell cortex part. In molecular function category, the genes were involved in guanylate kinase activity, WW domain binding, nucleotide kinase activity, phosphotransferase activity, phosphate group as acceptor, nucleobase, nucleoside, nucleotide kinase activity, microtubule motor activity and motor activity.

### Hub gene GSEA analysis

**Figure 8:**
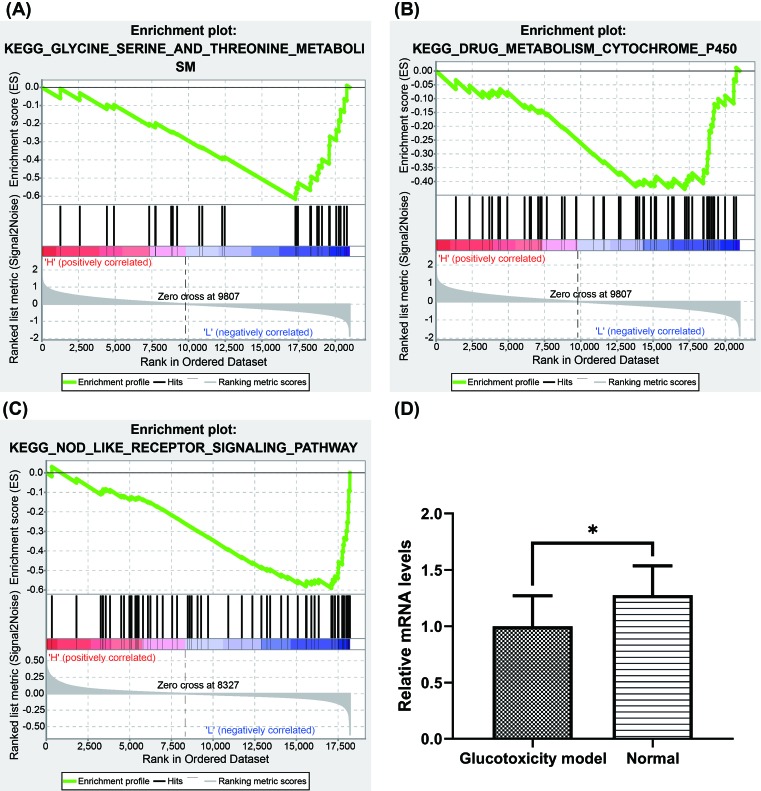
ACLY expression was associated with metabolic process and was validated by qPCR (**A,B**) GSEA analysis of low expression of ACLY in GSE20966. (**C**) GSEA analysis of low expression of ACLY in GSE38642. (**D**) ACLY mRNA levels in glucotoxicity model and normal group (*n*=5). Data are presented as mean ± S.D. *P<0.05 in glucotoxicity model vs normal group by Student’s *t* test.

To further clarify the possible mechanism of action of related genes in diabetes, we performed GSEA analysis on hub gene ACLY. According to the median of the gene expression value, we divided the samples into high expression group and low expression group. As shown in the Figure 8A–C, GSEA analysis suggests low expression of ACLY is enriched in glycine serine and threonine metabolism, drug metabolism cytochrome P450 (CYP) (GSE20966) and NOD-like receptor (NLR) signaling pathway (GSE38642) .

### The validation of hub gene expression

We performed qPCR in mouse glucotoxicity model *in vitro* to further verify the expression of hub gene. As illustrated in Figure 8D, compared with normal group, the transcription level of ACLY in glucotoxicity model group was significantly decreased (*P*<0.05), which is consistent with our bioinformatics analysis results above.

## Discussion

At present, the diagnosis and treatment of type 2 diabetes is far from satisfactory, and the incidence of this disease is still rising. Research on the pathogenesis and biomarkers of type 2 diabetes is still necessary. Exploring the molecular level dysfunction of islet cells of type 2 diabetes can provide effective treatment and more predictive and diagnostic biomarkers. For example, Jia et al. [12] identified marker genes for childhood-onset type 2 diabetes from one GEO dataset by performing DEGs, weighted co-expression network analysis (WGCNA), GO and KEGG enrichment, PPI network, diseases and transcription factors (TFs) related to critical genes analysis. Shao et al. [13] identified that the glucocorticoid was central in the pathogenesis of type 2 diabetes from three GEO datasets by integrated method including DEGs screen, gene functional enrichment, PPI network analyses, drug–gene interactions and genetic association of DEGs. Ni et al. [14] identified key genes in the diabetic wounds from one GEO dataset using a bioinformatics approach incorporating DEG screen, PPI network and phylogenetic analysis. Chen et al. [15] used the gene expression profiles from GEO datasets and identified candidate genes for type 2 diabetes and obesity by screening of genes, GO and pathway analysis. Here, we integrated three datasets from GEO and got 45 DEGs, the vast majority of which were down-regulated. The top 20 were *SLC2A2, ARHGEF9, EDN3, RRAGD, MAK16, RHOBTB3, HADH, TRIM37, CLGN, CTNNA2, ACLY, ABHD10, ATRNL1, ENTPD3, GRAMD3, CCL21, TGFBR3, NBEA, IAPP*. Ten genes were up-regulated, and they were *APOBEC2, FKBP2, GRHL2, ITGA3, PID1, PTGES, S100A14, S100A6, SAMSN1* and *THBS2*. Subsequently, we performed GO and KEGG functional enrichment on these DEGs. In addition, PPI were performed on DEGs. We investigated the module analysis of the PPI by MCODE and BINGO in Cytoscape. One of the hub gene was selected for further GSEA analysis. By performing with GO analysis, the DEGs mainly participated in protein localization, response to FSH, response to prostaglandin E and cell–cell signaling. These biological progresses are important processes involved in the pathophysiological mechanism of type 2 diabetes. A Hong Kong osteoporosis study showed that high-serum FSH was negatively correlated with diabetes [16]. Another showed that FSH enhances gluconeogenesis via CRTC2 and is correlated with fasting hyperglycemia [17]. Recent research suggested that prostaglandin E2 (PGE2) receptor EP3 is involved in water reabsorption then leads to abnormal renal function and polyuria in a mouse model of diabetes [18]. Neuman et al. [19] found that Eicosapentaenoic acid (EPA) treatment can enhance formation of PGE3 and reduce IL-11β expression in islet and improve β-cell function. KEGG pathway analysis revealed that DEGs are associated with the MODY. Mutation in gene level can cause MODY. TFs such as HNF4α (MODY1), HNF1α (MODY3), PDX1 (MODY4), HNF1β (MODY5) and NEUROD1 (MODY6) are often affected by this mutation. Mutations of TF lead to pancreatic islet cell metabolic abnormalities [20]. The results indicated that type 2 diabetes and MODY have some common molecular pathway.

We mapped a PPI program of DEGs. Then, MCODE and BINGO in the Cytoscape were performed. The genes in the MCODE were *DHRS2, ABHD10, HADH, ACLY*. ACLY is the hub gene. ACLY is a key molecule in celluar lipid production. It can convert cytosol into acetyl-CoA, which is essential for fatty acid synthesis. Depletion of ACLY protected cancer cells from hypoxia-induced apoptosis through modulating ETV4 via α-ketoglutarate. ACLY is not only involved in cancer cell signaling pathway, but also participates in the metabolism process of diabetes. Evidence indicated that ACLY could be inhibited by exogenous lipids and caused islet cell apoptosis and endoplasmic reticulum (ER) stress [21]. ACLY has been associated with a glucose-to-acetate metabolic switch [22]. Overall, ACLY in islet plays a key part in the molecular level of type 2 diabetes and maybe a potential target for diabetes.

The other three genes were *DHRS2, ABHD10* and *HADH*. HADH encodes short chain 3-hydroxyacyl-CoA dehydrogenase (SCHAD). SCHAD is important for β-oxidation of fatty acids when there is no glucose. Mutation of HADH can lead to increased pancreatic β-cells insulin secretion and contribute to blood glucose. As a result, this change can lead to leucine-induced hypoglycemia [23,24]. Although HADH plays an important part in HH, the molecular mechanisms in type 2 diabetes is still unknown currently and remains to be studied. DHRS2 belongs to the short-chain dehydrogenase/reductase family. It participates in many metabolic processes such as process in steroids, prostaglandins and xenobiotics [25]. ADHRS2 has been studied to be associated with esophageal squamous cell carcinoma and gastric carcinogenesis [26]. Currently, there have been no reports about the function of DHRS2 in type 2 diabetes. Further study about the relation between ADHRS2 and diabetes needs to be conducted. A study has reported that human ABHD10 is closely related to probenecid acyl glucuronidation in liver [27]. There is currently no report whether ABHD10 is involved in diabetes. Further work can be performed to investigate the potential role of ABHD10 in type 2 diabetes.

In biological process category performed by BINGO, the top five process the genes significantly enriched were lipid metabolic process, small molecule catabolic process, ATP catabolic process, citrate metabolic process and myeloid dendritic cell differentiation. These processes are also involved in type 2 diabetes. This is consistent with our previous metabolic processes involved in the genes of the module.

As mentioned before, we identified ACLY as hub gene and performed GSEA to deeply understand the molecular mechanism of ACLY in type 2 diabetes. GSEA analysis suggested low expression of ACLY correlated with glycine serine and threonine metabolism, drug metabolism CYP and NLR signaling pathway. Notably, these pathways are associated with the metabolic processes in type 2 diabetes.

Human glycine comes from glucose, betaine and threonine. Diabetes patients have lower serum glycine than healthy people. Serum glycine increases after improvement of insulin resistance [28]. In a study, hypoglycinemia predicts the risk of developing type 2 diabetes and elevated glycine level is correlated with a reduced risk of developing type 2 diabetes. At the same time, higher dietary glycine intake was associated with a reduced risk of pre-diabetes [29].

CYP enzymes are involved in the metabolism of drugs and xenobiotics. The CYP enzyme is the main catalyst for drug biotransformation reactions and is active in many chemical materials [30]. A study showed that combined therapy with insulin and aspartame in diabetic rats induced CYP2E1 in the brain, which could have toxicological effects [31]. Another study in India indicated the variable distribution of CYP2C8 and CYP2C9 allelic polymorphisms in people with diabetes [32]. Study showed that ER stress is involved in molecular process of type 2 diabetes. Inhibition of CYP4A inhibits hepatic ER stress. As a result, liver apoptosis and insulin resistance are reduced [33]. This study indicates a new relationship between CYP enzymes and type 2 diabetes.

NLRs are a group of pattern recognition receptors (PRRs) located inside the cell and are important for sensing pathogen and inflammation. NOD1 and NOD2 are well-characterized NLRs in the NOD signalosome. They can drive NF-κB signal pathway and lead to release of a series of cytokines. Intracelluar NLRs can further form inflammasomes with other molecules [34]. NLRP inflammasomes are well studied in type 2 diabetes. A study suggested that hyperglycemia can lead to activation of NLRP1 inflammasome and then contribute to neuron inflammation [35]. Another study indicated that decrease in NLRP3 expression in adipose tissue is associated with reduction in inflammation and improvement of insulin-sensitivity in type 2 diabetes with obese patients [36]. More and more studies showed that NLRP3 inflammasome plays a pivotal part in diabetic cardiovascular complications and diabetic retinopathy [37,38].

To further validate the importance of these key genes we have explored, we performed qPCR of hub gene ACLY *in vitro*. We found that the relative transcription level of ACLY showed the same expression trend as in our bioinformatics analysis. These genes, particularly ACLY, have had few researches on their effects on islet function in type 2 diabetes. Through our research, we thought that ACLY may affect islet β-cells through several pathways such as glycine serine and threonine metabolism, drug metabolism CYP and NLR signaling pathway. Whether these co-expressed genes affect the function of type 2 diabetes islet β-cells through the above pathways or other pathways provides a possible way of exploring the pathogenesis of type 2 diabetes. With further studies, these key genes may be expected to become new diagnostic and therapeutic biomarkers for type 2 diabetes.

There are some advantages in our research. First, to the best of our knowledge, our research is the first study to integrate all datasets related to human islets of type 2 diabetes in the GEO database for bioinformatics analysis. Second, the present study truly reflects the genetic-level changes in pancreatic β-cells during the pathophysiological process of type 2 diabetes and can provide possible target molecules for further research. Third, the methods of this research are easy to learn and can provide some ideas for disease data mining.

Because the sample size of the three datasets is not very large, and some pathogenic genes may not be significantly differentially expressed, the cut-off values we selected in the present study are relatively loose. Further experimental studies are needed to confirm the identified genes and pathways. With more uploaded GEO datasets, the development of related technologies and the improvement of algorithms, bioinformatics analysis will further promote the development of diabetes molecular pathogenesis and therapeutic research.

## Conclusion

For the first time, our study obtained 45 DEGs by integrating three sets of human diabetes islet datasets in GEO. Through PPI and GSEA, our study found that DHRS2, ABHD10, HADH and ACLY function together in type 2 diabetes. We identified the hub gene ACLY and the pathways involved. The present study provided useful information for further exploration of the pathogenesis of type 2 diabetes. In the future, more datasets are needed to be integrated to reduce the bias of the biometric analysis process. Further experiments *in vitro* and *in vivo* are needed to validate the role of these screened genes and pathways in the progression of type 2 diabetes.

## Abbreviations

ABHD10: α/β hydrolase domain 10
ACLY: ATP-citrate lyase
BINGO: Biological Networks Gene Ontology tool
CRTC2: Cyclic AMP-regulated transcriptional coactivator 2
CYP: Cytochrome P450
DAVID: Database for Annotation, Visualization and Integrated Discovery
DEG: Differentially expressed gene
DHRS2: Dehydrogenase/reductase 2
ER: Endoplasmic reticulum
FSH: Follicle stimulating hormone
GEO: Gene Expression Omnibus
GO: Gene ontology
GSEA: Gene set enrichment analysis
KEGG: Kyoto Encyclopedia of Genes and Genomes
MCODE: Molecular Complex Detection
MODY: Maturity onset diabetes of the young
NLR: NOD-like receptor
NLRP: NOD-like receptor protein
NOR: Nucleotide-binding oligomerization domain
PPI: Protein–protein interaction
qPCR: Quantitative real-time PCR
SCHAD: Short chain 3-hydroxyacyl-CoA dehydrogenase
STRING: Search Tool for the Retrieval Interacting Genes
TF: Transcription factor
